# Practice-informed implementation: a multi-method approach to map strategies for sustainable tobacco treatment integration within healthcare settings

**DOI:** 10.3389/frhs.2026.1749305

**Published:** 2026-08-04

**Authors:** Katy Ellis Hilts, Jyotsna Gutta, Annette McDaniel, Curtis Williamson, Natalie Rivich, Katelin Rupp, Miranda Spitznagle, Valerie A. Yeager

**Affiliations:** 1 Department of Health Policy & Management, Richard M. Fairbanks School of Public Health, Indiana University Indianapolis, Indianapolis, IN, United States; 2 Nicotine Dependence Program, Community Health Network, Indianapolis, IN, United States; 3 Tobacco Prevention and Cessation Commission, Indiana Department of Health, Indianapolis, IN, United States

**Keywords:** implementation blueprint, implementation strategies, strategy mapping, tobacco use, tobacco use cessation

## Abstract

**Background:**

Tobacco use remains the leading cause of preventable disease and death in the U.S., yet integration of evidence-based tobacco treatment in healthcare is often inconsistent and not sustained. To address this gap, there is a growing need for implementation strategies that are informed by real-world practice and tailored to local context. This study evaluates the implementation and sustainability of a tobacco treatment program within a large Midwestern health system, developed through a health systems change partnership with the Indiana Department of Health. Using a practice-informed approach, we applied multiple implementation science frameworks to retrospectively identify contextual factors influencing implementation and to map strategies across distinct implementation phases.

**Methods:**

We used a multi-methods design, integrating data from semi-structured interviews (*n* = 15), and the Clinical Sustainability Assessment Tool (CSAT). Interviews were guided by the Consolidated Framework for Implementation Research (CFIR) and analyzed thematically to identify determinants of implementation and sustainment. The CSAT was administered to core program staff to assess organizational capacity for long-term sustainability. Strategy mapping was conducted using the Replicating Effective Programs (REP) and Expert Recommendations for Implementing Change (ERIC) frameworks to sequence implementation strategies across four phases: pre-conditions, pre-implementation, implementation, and maintenance.

**Results:**

Interviews revealed iterative adaptations to the Nicotine Dependence Program, including EHR integration, virtual care expansion, and strategic workforce development. Key facilitators included leadership engagement, presence of a dedicated implementation leader, alignment with organizational culture, and integration into existing workflows. Barriers included limited reimbursement mechanisms and workforce constraints. CSAT results indicated high sustainability potential (mean score: 6.1/7), with strong ratings in outcomes and effectiveness, staff engagement, and monitoring and evaluation. Strategy mapping identified over 20 discrete strategies across REP phases, offering a replicable blueprint for other systems.

**Conclusions:**

Practice-informed, context-responsive implementation can lead to sustainable systems change for tobacco treatment. Findings highlight the importance of inner setting factors, including leadership support, workflow compatibility, and culture of continuous improvement, in sustaining evidence-based interventions. This study provides a model for drawing evidence from real-world implementation to identify strategies which can serve as a guide for health systems seeking to integrate and sustain evidence-based tobacco treatment services.

## 1 Introduction

Tobacco use remains the leading cause of preventable disease and death in the U.S., with approximately 46 million adults reporting current use in 2021 (1, 2). Despite strong evidence supporting the effectiveness of tobacco treatment interventions, integration of services into routine healthcare delivery remains inconsistent and often not sustained (1, 3). The U.S. Public Health Service Clinical Practice Guidelines for Treating Tobacco Use and Dependence emphasizes the importance of systems change strategies, defined as modifications to healthcare infrastructure, workflows, and policies, to embed tobacco treatment into standard care (4). Systems change interventions for tobacco treatment involve routine identification of tobacco users, delivery of evidence-based treatments, and documentation within electronic health records (EHRs) (4). However, widespread implementation of such systems-level interventions is often limited by organizational and operational barriers including misaligned workflows, lack of leadership prioritization, and insufficient staff training (5, 6).

To advance the integration of evidence-based interventions, such as tobacco treatment, into healthcare practice, there is a need to identify implementation approaches that are practice-driven and contextually grounded (7, 8). Practice-informed implementation research, which enables researchers and practitioners to collaborate closely within the context of real-world implementation, offers a model for this shift (9, 10). This approach supports a deeper understanding of local context and supports the identification of strategies that are both feasible and sustainable (7, 9, 10). Practice-informed evidence, generated through qualitative inquiry and grounded in real-world implementation, enables tailoring of interventions to support integration in healthcare settings (8, 9). By learning from successful, practice-based implementation within healthcare settings, we can better understand how to operationalize interventions, adapt strategies to fit local workflows, and ultimately improve the reach and impact of evidence-based interventions, such as tobacco treatment services. This inside-out perspective re-centers the expertise of frontline providers and health system leaders, ensuring that implementation efforts are not only evidence-informed but also practice-relevant (7, 11).

**Figure 1:**
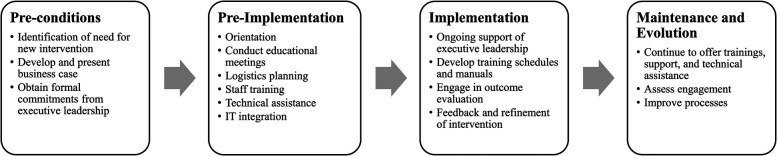
Replicating effective programs (REP) framework for implementation of tobacco treatment interventions in healthcare settings. This figure groups strategies by four distinct phases of implementation: pre-conditions (activities/factors influencing decision to implement an evidence based practice(EBP)), pre-implementation (activities/strategies used to prepare to implement EBP), implementation (activities/strategies used during the implementation of the EBP), and maintenance (activities/strategies used to support the ongoing delivery and sustainment of the EBP).

As part an ongoing evaluation of a health systems change partnership between the research team, Indiana Department of Health, and a large health system in Indiana, this study sought to evaluate the implementation and sustainment of a comprehensive tobacco treatment program. Using a multi-methods design, we retrospectively mapped implementation strategies to stages of implementation, drawing on the work of Huynh, et al. to operationalize and sequence strategies across the implementation continuum (12). In the referenced article, Huynh and colleagues present a pragmatic approach to map strategies to support implementation of evidence-based interventions utilizing the Replicating Effective Programs (REP) framework. This framework, illustrated in Figure 1, groups strategies by four distinct phases of implementation: pre-conditions *(activities/factors influencing decision to implement an evidence based practice(EBP)*), pre-implementation (*activities/strategies used to prepare to implement EBP*), implementation (*activities/strategies used during the implementation of the EBP*), and maintenance (*activities/strategies used to support the ongoing delivery and sustainment of the EBP*) (13, 14). By utilizing this approach in the current study, we aimed to generate practice-informed evidence and create an implementation blueprint to support other health systems seeking to develop sustainable tobacco treatment services. By taking an inside-out rather than outside-in approach, this work contributes to the growing call for integrated, context-responsive approaches to advancing the implementation of evidence-based care (10, 11).

## 2 Methods and materials

### 2.1 Intervention implementation and setting

In 2018, the Indiana Department of Health's Tobacco Prevention and Cessation Commission (IDOH TPC) established a health systems change partnerships to support capacity building and the development of system-level solutions to address tobacco use among health system's patients through the integration of tobacco dependence treatment into routine practice (15). Through this grant program, IDOH TPC and its partners provided technical assistance and services related to (1) implementing best practices for tobacco dependence treatment, with a focus on care coordination, (2) quality improvement, and (3) utilization of electronic health record systems to support tobacco treatment.

The research team has provided ongoing evaluation for this work since the grant's inception. The current study focuses on the successful and sustained implementation of a tobacco treatment program within Community Health Network (CHNw), a large non-profit, multiple hospital, health system in the Midwest serving the Indianapolis metropolitan area and surrounding rural counties. CHNw's Nicotine Dependence Program (NDP) began as a pilot program, in 2017, serving the inpatient and outpatient population in the network's cardiovascular service line. CHNw received its initial IDOH TPC Health Systems Change grant in 2018 with continuation of the grant award to through 2026. This partnership created funding, networking opportunities, and technical support to advance integration of evidence-based tobacco treatment strategies within CHNw. Since 2018, the partnership has fostered expansion of evidence-based tobacco treatment across the health system.

The CHNw NDP provides free treatment (i.e., they do not bill for services) for patients and employees for all types of tobacco products (including e-cigarettes). The NDP is staffed by highly trained clinicians including physicians, nurse practitioners, pharmacists, registered nurses, and tobacco treatment specialists (TTS). The program consists of eight virtual, individualized visits with a program duration of approximately 12 weeks (see Supplementary Figure S1 in Supplemental Materials). During the initial six years of the program, 2019–2025, the NDP served 4,060 unique individuals (employees and patients) with over 19,000 visits. During this time, the patient program completion quit rate was 56% and the employee program quit rate was 60%.

### 2.2 Study design

We employed a multi-methods design, combining qualitative interview and quantitative survey data, to comprehensively evaluate the implementation and sustainability of the CHNw NDP. Qualitative data was collected through semi-structured interviews, while a validated survey, the Clinical Sustainability Assessment Tool (16), was used to collect quantitative data related to sustainability. Qualitative and quantitative data were analyzed independently through qualitative coding and descriptive statistics to identify common factors influencing implementation and sustainability of the program, then combined to inform the strategy mapping process. All study procedures were reviewed and approved by the Indiana University Institutional Review Board and Community Health Network's Institutional Review Board. Prior to participation, all interviewees and assessment respondents provided informed consent. Data were securely stored on encrypted, access-controlled platforms in compliance with institutional data protection guidelines. Full study procedures are described below.

### 2.3 Participant recruitment and sampling

We used purposive sampling to identify individuals with varied roles and perspectives on the CHNw NDP, including program designers, the implementation lead (director of the NDP), clinical staff, administrative leaders, technical assistance partners, and frontline implementers (see Supplementary Table S1 in Supplemental Materials). A total of 15 participants were recruited and participated in one-on-one interviews, which was sufficient to achieve thematic saturation (17). Recruitment was facilitated through internal referrals, program contacts, and snowball sampling to ensure a broad representation of perspectives. Additionally, to evaluate the program's perceived sustainability from the vantage point of frontline deliverers, five TTSs, all of whom were part of the core NDP staff with involvement in the implementation or delivery of the program, were recruited and completed the sustainability assessment.

### 2.4 Interview procedure and framework

Interviews were conducted by trained members of the research team via secure videoconferencing platforms (e.g., Zoom). Interviews followed semi-structured interview guides (included in Supplementary Materials) tailored to role and grounded in the Consolidated Framework for Implementation Research (CFIR) (18). CFIR is a determinants framework that includes constructs from multiple implementation frameworks and theories providing a comprehensive structure to assist in explaining barriers and facilitators of implementation. The interview guide was adapted and refined iteratively by the research team. Questions were mapped to key CFIR constructs but retained flexibility to allow participants to elaborate on emergent themes or describe events in their own terms. All interviews were audio-recorded with permission and transcribed verbatim.

### 2.5 Interview analysis and coding procedures

De-identified transcripts were uploaded to Dedoose for qualitative data management and analysis. The coding of transcripts was guided by the CFIR. In our analyses, constructs from CFIR were organized across three primary domains: (1) innovation characteristics, (2) implementation process, and (3) inner setting. Additionally, the research team included codes for facilitators, barriers, and strategies to inform the strategy mapping process described below.

The coding process was conducted in several structured stages. Initially, three team members with qualitative experience independently coded a shared subset of interviews to establish inter-coder reliability and refine a common codebook. This was followed by consensus meetings to resolve discrepancies and ensure consistent interpretation of CFIR constructs. Once calibrated, the remaining transcripts were coded in dyads, with each pair discussing and resolving uncertainties collaboratively. Throughout the process, the full team met periodically to review themes, verify coding consistency, and confirm alignment with theoretical frameworks.

### 2.6 Sustainability assessment

To complement qualitative findings with structured data on long-term feasibility, the five TTSs who make up core program staff were invited to complete the Clinical Sustainability Assessment Tool (CSAT). The CSAT is a validated survey instrument designed to assess the extent to which clinical interventions are supported for long-term sustainability (16). It encompasses 42 items distributed across seven domains: *engaged staff and leadership, organizational readiness, workflow integration, monitoring and evaluation, implementation and training, champions and advocacy,* and *program adaptability.*

TTSs completed the CSAT individually via a secure electronic platform provided by the Center for Public Health Systems Science at Washington University in St. Louis. Responses were analyzed descriptively through the system and compared with thematic findings from interviews to assess convergence and divergence in perceived sustainability across different implementation dimensions.

### 2.7 Iterative timeline mapping by implementation phase

Following interview and CSAT analysis, we conducted the iterative mapping process to construct a timeline of implementation strategies and activities organized by REP phase: pre-conditions, pre-implementation, implementation, and maintenance (see Figure 1). This process included a review of internal documents and coded interview narratives to capture the sequence, nature, and context of program activities. Interview data were integrated to fill gaps and add narrative depth. Participants provided nuanced insights about informal processes, course corrections, and critical incidents that were not captured in formal documentation. To guide the identification of implementation strategies, we applied the Expert Recommendations for Implementing Change (ERIC) framework (19). ERIC includes 73 defined implementation strategies grouped into thematic categories.

This was followed by a review of archival documents which were selected purposively in collaboration with the implementation lead based on relevance to key implementation milestones. The review was used to confirm timing and sequence of formal milestones such as training dates, protocol releases, and program launches, and as such did not undergo formal coding. Documents reviewed included business and implementation plans, clinical policies and protocols, workflow diagrams, medication algorithms, quality improvement materials, and staff training resources related to the development and implementation of the NDP (see Supplementary Table S1 in the Supplemental Materials).

As described above, during the analysis of interview data, the team identified barriers, facilitators, and strategies related to the implementation of the NDP. This was used to extract relevant excerpts to refine and contextualize timeline elements. Timeline development and strategy mapping was iterative, and team based. Preliminary versions were reviewed in analytic meetings and revised through consensus. The team involved in this process included three members of the research team and the director of the NDP (*implementation lead*). The inclusion of the implementation lead and review of documents assisted in confirming the accuracy of identified strategies and timeline of activities.

## 3 Findings

### 3.1 Qualitative interviews

Below we present the results of our qualitative analyses, with themes organized by overarching CFIR construct for which there was data: *Innovation Characteristics, Implementation Process, and Inner Setting*. We include representative quotes for each theme within the text to support the findings and align activities identified by interviewees with ERIC strategies presented in [brackets]. We interviewed 15 individuals representing hospital leadership, dedicated NDP staff, internal technical assistance (TA) (information technology, quality improvement), external TA, referring providers, and NDP champions (pharmacy, behavioral health, cardiology). We note the role of the interviewee after each quote and Supplementary Table S2 in Supplementary Files provides the number and types of interviewees along with a description of roles.

#### 3.1.1 Innovation characteristics

##### 3.1.1.1 Trialability

As defined by CFIR, trialability refers to the ability to test an innovation on a small scale prior to implementing it more broadly across an organization. Interview participants noted that the NDP being piloted at a smaller scale supported the expansion of the program [*stage implementation scale up*]. As one interviewee stated,

“So, it was initially piloted with a very small patient population in just one region. And then as we developed something that was thought to be sustainable enough to expand it, more staff was brought on board and some further education efforts, so that we could have more boots on the ground and start to integrate the delivery of this care in other regions and across other product lines within the organization”—(Dedicated NDP Staff)

While the trialability of the NDP was particularly important to support the initial proof of concept, participants also noted that the NDP had piloted new services at a smaller scale [*conduct cyclical small tests of change*] as part of planning for expansion of the program to other parts of the health system. The ability to do this was seen as an important way to determine what did and did not work to ensure that resources were used appropriately before wider dissemination.

##### 3.1.1.2 Relative advantage

The NDP was perceived to be superior to other options available, or previous tobacco initiatives that had been tried (e.g., referrals to the state's quitline). Interviewees emphasized that the NDP was developed as a response to the absence of standard clinical and evidence-based tobacco cessation programming within the system. Additionally, interviewees identified that opportunities existed for greater collaboration between clinical departments and that having a single consistent program [*create new clinical teams*] within the system reduced patient siloing and duplicative services, making this a key advantage of the NDP.

“I think, having a true … structured program rather than just the generic 1–800 number, you know … giving it a little more tangibility was probably the biggest thing that we needed.” –(Referring Provider)

##### 3.1.1.3 Adaptability

Adaptability was seen as another facilitator to the NDP's success. Interview participants noted that the ability of the program to adapt as it grew (e.g., removing in-person requirements) [*promote adaptability; intervene with patients/consumers to enhance uptake and adherence*] allowed more participants to engage in the program, and to overcome barriers such as transportation to meet with NDP practitioners and engage in cessation counseling. Some of the programmatic adaptations were due to the COVID-19 pandemic, but interview participants indicated that these changes, incorporated into the program protocols, aided in patient engagement and enhanced the reach of the NDP.

“The initial protocols were written that the very first [visit] had to be in person, and we did see that as a barrier for some … And then as we were growing and that was evolving, and we were realizing we were ready to expand, but we have this barrier with geography in such a large network … along came the COVID-19 pandemic and it demanded that we … look at that challenge. The protocols were adjusted to allow for all virtual care and that has been a very good thing in most cases. It allows us to serve more people. It eliminates many of the barriers in terms of transportation and things that people encounter with in person visits.”—(Dedicated NDP Staff)

#### 3.1.2 Implementation process

##### 3.1.2.1 Planning

The planning phase per CFIR includes the degree to which roles and responsibilities are identified, specific steps and milestones are laid out, and goals and measures of success are defined. Key activities noted in the planning phase for the NDP included developing standardized training and education materials [*develop educational materials*], training TTSs [*make training dynamic; conduct educational meetings*] and integrating new processes into the EHR [*change record systems*] with information technology (IT) support.

Interviewees noted that while the process was complicated, the use of multidisciplinary teams [*use advisory boards and workgroups*] and being able to secure executive leadership [*obtain formal commitments; mandate change*] support early on were critical to the success of the implementation process.

“It was complicated, mainly because we wanted to get everything done correctly. And so, you know, the first thing obviously, is getting the engagement at the high-level executive team and then you needed these cross-functional teams … we had an overwhelming amount of projects they had to do so it took us some time to prioritize … and then hiring people and getting the finances, there were a lot of barriers; having [Executive Leader support] helped with those barriers.”—(Hospital Leadership)

##### 3.1.2.2 Opinion leaders

Interviewees emphasized that having the right people on board from the start supported the successful implementation of the NDP. The formation of a multidisciplinary team [*use advisory boards and workgroups*], including Vice Presidents of product lines, IT, finance, and other essential personnel, including physicians and Advanced Practice Providers (APPs), was vital for planning and executing the program. Further, it was noted that identifying the NDP as a network priority [*mandate change*] helped gain the buy-in of key individuals within the organization, supporting the program's expansion.

“I mean, we had a great physician champion engagement across the board … I feel like once that executive network team said, “Hey, this is going to be a priority for us,” you have all these other areas that are reporting the quality outcomes, physicians socializing the program with their peers … So, I feel like there's good support.”—(Internal TA)

##### 3.1.2.3 Formally appointed internal implementation leaders

Interviewees highlighted the importance of the implementation leader's passion and commitment to addressing tobacco use and improving patient outcomes in the success of the NDP. Interviewees also noted the importance of executive leadership's commitment to the creation of a formal position to lead the implementation [*revise professional roles; identify and prepare champions*] and ongoing support of the program. While interviewees acknowledged that the implementation did face challenges along the way, they felt that having an individual solely dedicated to the program facilitated the successful implementation.

“So, we had our board support, our executive support, and then [the implementation leader] became the program director. From there she's been trailblazing the work … So, I applaud our network leaders for giving her the opportunity to implement this program here because I don't know if anyone else in our local [health] systems has anything like this.”—(Internal TA)

##### 3.1.2.4 Champions

In addition to the formal implementation leader, interviewees highlighted many champions [*identify and prepare champions]* who were crucial to the successful development and implementation of the NDP. It was noted that champions spanned many points in the development of the program and encompassed different areas of the network. Further, engaging individuals across the organization [*promote network weaving*] with a drive to support patients in quitting tobacco has allowed for continued growth and expansion of the NDP.

“I have appreciated the amount of diversity that we've brought to the team and the fact that we're starting to branch out across various departments. I think it's going to make the program, even more well-known so more patients will get help … Everyone that's involved seems to be passionate about it. I love that it's not people being volunteered into the role; they're asking for people who are passionate about it. I think that comes through for the program as well as for the patients.”—(Referring Provider)

##### 3.1.2.5 External change agents

In addition to internal support, the development and implementation of the NDP was facilitated through partnership with external groups [*use an implementation advisor; shadow other experts*], such as IDOH TPC and MD Anderson's Tobacco Treatment Program. Several interviewees highlighted the partnership with MD Anderson, which supported tobacco treatment specialist training, including a local, onsite opportunity, and assistance in standardizing training materials and processes. Interviewees also noted that they had been able to tap into ongoing technical support both directly and as part of a national network of others working in tobacco treatment.

“I know MD Anderson … they’ve been super supportive … we modeled our program after them and asked them questions along the way. I got trained [as a TTS] through MD Anderson. A lot of us go to their ECHOs that they have a couple of times a month … So, it's clear there's a close partnership and I would say they've been the biggest outside organization that has supported the work that we've done.”—(Dedicated NDP Staff)

##### 3.1.2.6 Reflecting and evaluating

The last process-related theme centered on how data was being tracked and used to inform the NDP. Interviewees discussed the development and importance of a data dashboard for tracking data [*develop and organize quality monitoring systems*], both formally and informally, to inform program progress and make changes to existing processes [*audit and provide feedback*]. It was also noted that the various teams involved in implementing the NDP regularly use this data in meetings to inform ongoing process improvement activities. Interviewees noted the importance of using the data to identify ways to better support patients and ensure they are reaching those most in need of the services.

“And there's a dashboard where we can view our personal and department results. So, we can see how we're doing and if anything, that we're doing training wise or as a department, any of that is helping improve the care that we're providing to the patients.”—(Referring Provider)

#### 3.1.3 Inner setting

##### 3.1.3.1 Culture

Common values within the system around caring, supporting, and holistically addressing the needs of patients were seen as key drivers of the success and sustainability of the NDP. Interviewees consistently discussed a “patient-centered” culture aimed at informing and improving practice.

“So, the patient being first and foremost but also [the network] realizes that, depending on demographics … you have to be more creative and honest with patients in trying to meet them wherever they are based on their obstacles … I think that's part of how the NDP has figured out how to be successful … not allowing those types of things to be obstacles to providing the care.”—(Referring Provider)

In addition, interviewees emphasized the commitment from system leadership [*mandate change*; *obtain formal commitments*] aimed at reducing barriers and promoting tobacco treatment by eliminating the costs of the NDP to recipients, being open to practice-informed feedback, and including employees as recipients of the NDP as key facilitators to the successful implementation.

“The network as a whole, we're not directly billing; we're not directly recouping a lot of that financial side of it, but they know that this is going to benefit the community. And so, the fact that we've gained that kind of support … We know that we're going to improve health, improve quality of life, and prevent a lot of long-term issues downstream if we target this now.”—(NDP Champion)

##### 3.1.3.2 Implementation climate

Interviewees broadly perceived that the NDP was endorsed by the system and seen as a major priority [*mandate change*], which led to a supportive implementation climate across the system. Support provided for implementation and sustainment came from leadership across multiple areas of the system [*involve executive boards*], including hospital leadership, clinical departments (including pharmacy, behavioral health, surgery, cardiology), and IT.

“It was one of the network initiatives. And I know that means that everyone's volunteering for it. Everyone's prioritizing it. And so, even though you only get that for a certain period of time, my feeling is that those projects still continue to be well valued even once the spotlight time is over.”—(Referring Provider)

Further, many noted that those implementing the NDP were empowered to think creatively and provide feedback to leadership on ways that the innovation could be improved [*promote network weaving; inform local opinion leaders*] and to enhance acceptance among clinicians and recipients.

“It's embedded in our thinking that we all own process improvement, and that anyone can speak up. Can we see what's being done right now that we want to improve on? And then that individual has ownership of that, and we'll work on it with a team outside of a meeting. So, it's kind of baked into our culture of continuous process improvement and has evolved over the last 5 years.”—(Dedicated NDP Staff)

##### 3.1.3.3 Leadership engagement

Leadership engagement was seen by interviewees as a crucial component of the successful implementation and sustainment of the NDP. Interviewees noted that several executive leaders were major proponents of the NDP and engaged in strategic decision-making that advanced its implementation. Further, it was noted that leadership prioritized the NDP as a part of the system's larger organization goals, negotiating for resources in funding, workforce, IT support and integration into clinical care [*obtain formal commitments; mandate change*].

“Due to the support from leadership, it has been easier than I have seen other programs be in other places … Cost and sustainability is always a challenge and always will be but the, the network is committed to continuing to explore the best way to make it happen … there’s an obvious real commitment to continuing the work…”—(Dedicated NDP Staff)

##### 3.1.3.4 Compatibility

Interviewees discussed several ways in which the NDP fit into the existing workflows of the system. These facilitators included: integrating NDP referrals and documentation into the EHR [change record systems], imbedding NDP clinicians into the offices/clinics they already work for [revise professional roles] and offering NDP specific training to clinicians across the system [*conduct educational meetings*]. Strategies were in place to integrate NDP into existing processes and avoid clinician burnout.

“We did not want to put any onus on already burnt-out clinicians, or even if they're not burned out there's so many things the clinician will have to do in an office visit that the easier, we could make it [the better]. So, referral in our EMR was one of the ways that we implemented that. Now that we've [expanded], some of our [NDP] clinicians are embedded in the offices where they work. For example, within our primary care office, our pharmacists are the clinicians that will be treating.”—(Dedicated NDP Staff)

##### 3.1.3.5 Access to knowledge and information

Education about tobacco treatment and the NDP was viewed as a key facilitator to increase access to knowledge and information. Several interviewees discussed key strategies to ensure knowledge and information about the NDP was accessible and readily available. These strategies included: creating specialized NDP training opportunities for system clinicians through technical partners [*use implementation advisor*], developing a quarterly newsletter for clinicians and stakeholders within the system [*inform local opinion leaders*], and producing a widely available training manual [*develop educational materials*]. NDP clinicians also promoted open channels of communication for clinicians seeking guidance on tobacco treatment and the NDP.

“We've aimed to send people for certified tobacco treatment training … and then standardizing materials so that people have guidance on how to deliver the care in alignment with evidence-based practice. And, of course, a lot of teams [were] involved to get those materials developed, to get approval for them, and to have them distributed and available to all in electronic form…”—(Dedicated NDP Staff)

##### 3.1.3.6 Available resources

Stakeholders generally felt that resources and support to meet the needs of the NDP recipients and practitioners were available across the system. However, a constrained workforce, largely due to limitations on how tobacco treatment services are billed, and physical space limitations were consistently identified as barriers to sustainment and growth of the program. Despite this, it was noted that the standardized protocols [*develop a formal implementation blueprint*] and EHR infrastructure [*change record systems*] facilitated the likely sustainment of the NDP in the long-term.

“I think the biggest challenge is in manpower and sustainability in terms of cost…that's, just the bottom line of it…. there are a lot of clinicians that can deliver this care and do it well … a lot of its rooted in-patient education, supporting people and obtaining resources like medications, making sure they don’t encounter barriers with getting those medications and then just having support with any challenges that they encounter when implementing their plan. And so, there are a lot of professionals that can assist with that work, but it's not always reimbursed, so the sustainability piece becomes difficult.”—(Dedicated NDP Staff)

### 3.2 Clinical sustainability assessment tool results

Responses to the CSAT were collected from five participants, and domain scores were averaged to evaluate the extent to which the program has institutional structures and processes supporting its long-term sustainability (see Table 1). The overall sustainability score was 6.1 on a 7-point scale. The *Outcomes and Effectiveness* domain received the highest rating (6.6), with all but one domain indicator receiving ratings of 6.8 or 7.0. The perceived cost-effectiveness of the program received the lowest rating (5.6) in this category. *Engaged Staff and Leadership* followed closely (6.5), with high ratings for team-based collaboration (7.0) and clinical champions (6.6). *Organizational Readiness* was the lowest-rated domain (5.4), with resources (5.0), (i.e., time, space, and funding) and staffing (5.4) receiving lower ratings, while respondents gave high ratings for strong cultural fit (6.6).

**Table 1:** Average sustainability rating by domain and domain indicator.

Domain/Indicator	Rating^a^
Overall Sustainability	6.1
Engaged Staff and Leadership	6.5
1. The practice engages leadership and staff throughout the process.	6.2
2. Clinical champions of the practice are recognized and respected.	6.6
3. The practice has engaged, ongoing champions.	6.2
4. The practice has a leadership team made of multiprofessional partnerships.	6.4
5. The practice has team-based collaboration and infrastructure.	7.0
Engaged Partners	6.0
1. The practice engages the patient and family members as partners.	5.0
2. There is respect for all partners involved in the practice.	6.4
3. The practice is valued by a diverse set of interested parties.	5.8
4. The practice engages other medical teams and community partnerships as appropriate.	6.0
5. The practice team has the ability to respond to feedback about the practice.	6.8
Organizational Readiness	5.4
1. Organizational systems are in place to support the various practice needs.	5.2
2. The practice fits in well with the culture of the team.	6.6
3. The practice has feasible and sufficient resources (e.g., time, space, funding) to achieve its goals.	5.0
4. The practice has adequate staff to achieve its goals.	5.4
5. The practice is well integrated into the operations of the organization.	5.0
Workflow Integration	5.9
1. The practice is built into the clinical workflow.	6.0
2. The practice is easy for clinicians to use.	5.8
3. The practice integrates well with established clinical practices.	6.0
4. The practice aligns well with other clinical systems (e.g., EMR).	6.2
5. The practice is designed to be used consistently.	5.6
Implementation & Training	5.9
1. The practice clearly outlines roles and responsibilities for all staff.	6.2
2. The reason for the practice is clearly communicated to and understood by all staff.	6.0
3. Staff receive ongoing coaching, feedback, and training.	5.8
4. Practice implementation is guided by feedback.	5.6
5. The practice has ongoing education across professions.	6.0
Monitoring & Evaluation	6.3
1. The practice has measurable process components, outcomes, and metrics.	7.0
2. Evaluation and monitoring of the practice are reviewed on a consistent basis.	6.2
3. The practice has clear documentation to guide process and outcome evaluation.	6.0
4. Practice monitoring, evaluation, and outcomes data are routinely reported to the clinical care team.	6.2
5. The practice process components, outcomes, and metrics are easily assessed and audited.	6.2
Outcomes & Effectiveness	6.6
1. The practice has evidence of beneficial outcomes.	6.8
2. The practice is associated with improvement in patient outcomes that are clinically meaningful.	6.8
3. The practice is clearly linked to positive health or clinical outcomes.	7.0
4. The practice is cost-effective.	5.6
5. The practice has clear advantages over alternatives.	6.8

a Scale of 1 to 7, where 1 = program has to no extent and 7 = program has to the full extent.

### 3.3 Implementation strategy mapping and timeline

In Table 2, we present strategies along with the goal and barrier it was designed to address during implementation. These strategies are then mapped to the REP implementation phase and ERIC strategies, with a description of the key activities involved. Table 3 provides ERIC strategies by REP phase and provides the estimated timeframe for each phase. Below we highlight strategies that were noted as key to the success of the program by the implementation lead during the iterative mapping phase.

**Table 2:** Nicotine dependence program (NDP) strategies and activities mapped to ERIC^a^ strategies by implementation phase .

NDP Strategy	Implementation Phase	ERIC Strategy and NDP-Specific Activities
Build the business case for NDP	Pre-condition	Assess for readiness and identify barriers and facilitators - Conduct internal interviews to obtain historical and current tobacco work within the organization Identify and prepare champions - Identify local experienced clinical mentor - Identify network sponsors for internal networking and championing the project
Goal: Gain approval to implement an NDP pilot.		
Barrier: Difficulty proving return on investment of NDP.		
Consult with experts who have experience in implementing the NDP	Pre-condition	Revise professional roles - TTS^b^ Certification of Implementation Leader Shadow other experts - Conduct conversations with experienced technical assistance nicotine dependence center Use an implementation advisor
Goal: Learn from experienced people familiar with successful NDP implementation.		
Barrier: Avoid common pitfalls and barriers to developing NDP within health systems		
Identify the NDP as a system priority to gain buy-in of key individuals	Pre-condition	Mandate change - Present business case to network executive leadership and receive approval Obtain formal commitments - Collaborate with hospital leadership and finance to pitch idea for pilot - Develop business case
Goal: Support program expansion		
Barrier: Scalability issues		
	Pre-implementation	Conduct educational meetings - Present NDP to referring physicians and office staff Stage implementation scale up - NDP Pilot is approved - Build NDP into EHR
Prioritize NDP as a part of the system's larger organizational goal	Pre-condition	Mandate change - Hospital leadership approves pilot
Goal: Advance implementation of NDP	Pre-implementation	Mandate change - Executive leadership approves the NDP to scale and prioritize project and allocates resources Stage implementation scale up - Executive leadership determines to scale the pilot to employees
Barrier: Resistance to rollout of NDP		
Utilize leadership across multiple systems (board of directors, executive/senior leadership, pharmacy, behavioral health, surgery, cardiology)	Pre-condition	Involve executive boards - Hospital leadership approves pilot Prepare champions - Identify project champions to support standardizing process and to ensure the implementation leader had the right people to inform
Goal: Increase clinician acceptance of NDP across the system.		
Barrier: Resistance to change or lack of buy-in from clinicians.	Pre-implementation	Promote network weaving - Executive leadership prioritizes work to allocate IT resources, project manager, and supports staff time to work on project
	Implementation	Inform local opinion leaders - Launch new program and present to clinical teams to promote referrals and market our employee program
	Maintenance and Evolution	Inform local opinion leaders - Launch new program and present to clinical teams to promote referrals and market our employee program
Form multidisciplinary team of VPs of product lines, IT, finance, and essential medical personnel	Pre-implementation	Develop a formal implementation blueprint - Executive leadership prioritizes work to allocate IT resources, project manager, and supports staff time to work on project Promote network weaving - Utilize network process improvement team to lead development of implementation plan
Goal: Support successful planning for implementation of NDP		
Barrier: Lack of strategic planning		
Negotiate resources for funding, workforce, IT support	Pre-implementation	Access new funding - Executive leadership prioritizes work to allocate IT resources, project manager, and supports staff time to work on project; - Executive leadership identifies and earmarks funds to support dedicated NDP staff to lead initiative Use advisory boards and workgroups - Create multidisciplinary team and create IT infrastructure, standard care process
Goal: Advance implementation of NDP		
Barrier: Resistance to rollout NDP		
	Implementation	Tailor strategies - Allocate resources for dashboard - Educate staff on how document in EHR
Integrate NDP referrals/ documentation into the EHR	Pre-implementation	Change record systems - Multidisciplinary team works with IT to create standard EHR documentation, data collection/dashboard Promote adaptability - IT imbeds clinical documentation tools
Goal: Integrate NDP into existing processes		
Barrier: Lack of cohesion between new and existing processes.		
	Implementation	Promote adaptability - Train clinicians to use documentation tool and provide ongoing technical assistance
	Maintenance and Evolution	Promote adaptability - Train clinicians to use documentation tool and provide ongoing technical assistance
Reduce inefficiencies and implement process improvement	Pre-implementation	Develop and organize quality monitoring programs - Multidisciplinary team works with IT to create standard EHR documentation, data collection/dashboard
Goal: Improve NDP processes.		
Barrier: Inefficiencies or outdated methods that hinder productivity.	Implementation	Promote adaptability - Multidisciplinary team evaluates current process and adjusts to fit needs Purposely reexamine the implementation
	Maintenance and Evolution	Promote adaptability - Evaluate outcome data and improve processes Purposely reexamine the implementation
Develop standardized training and education materials	Pre-implementation	Develop educational materials - Produce a widely available and comprehensive training manual - Develop different modalities to deliver training materials Make training dynamic - Multidisciplinary team collaborate with external organization and creates clinician training/educational materials - Train clinicians as TTSs
Goal: Ensure knowledge and information about the NDP is readily available		
Barrier: Information silos		
	Implementation	Develop educational materials - Develop educational presentation to deliver in-person to clinicians - Develop a comprehensive training manual Make training dynamic - Collaborate with external partners to create on-demand trainings for clinicians
	Maintenance and Evolution	Conduct educational meetings - Continue to offer on demand and in-person trainings - Support and provide technical assistance to newly trained TTSs Make training dynamic
Imbed NDP clinicians into offices/clinics they already work for	Pre-implementation	Create new clinical teams - Train staff on new referral process and educate on program Revise professional roles - Identify clinicians to deliver intervention
Goal: Avoid clinician burnout		
Barrier: High workload and stress levels among clinicians.		
	Maintenance and Evolution	Revise professional roles - Support and provide technical assistance to newly trained TTS - -Provide outcome data to referring office
Offer NDP specific training to clinicians across the system	Pre-implementation	Conduct educational meetings - Train clinicians to be TTS Conduct educational outreach - Visit clinical sites to educate on innovation
Goal: Integrate NDP into existing processes		
Barrier: Lack of streamlined operations		
	Implementation	Conduct educational meetings - Collaborate with external partners to create on demand trainings for clinicians Conduct educational outreach - Develop educational presentation to deliver in-person to clinicians - Support and provide technical assistance to newly trained TTS
	Maintenance and Evolution	Conduct educational meetings - Create internal collaborative group to present didactic educational material and case study presentations Conduct educational outreach - Support and provide technical assistance to newly trained TTS
Identify opportunities to expand and sustain program.	Pre-implementation	Conduct cyclical small tests of change - Idea was presented to hospital leadership to pilot innovation/intervention
Goal: Promote program growth		
Barrier: Funding constraints	Implementation	Stage implementation scale up - Present outcomes of pilot to executive leadership and approval to scale
	Maintenance and Evolution	Stage implementation scale up - Identify opportunities and needs to embed tobacco treatment in current care delivery process in departments/offices
Collect and interpret data supported by the EHR	Pre-implementation	Develop and organize quality monitoring systems - Create data dashboard
Goal: Guide decision-making to inform expansion or contraction of services		
	Implementation	Audit and provide feedback Facilitate relay of clinical data to providers - Collect data via EHR and dashboard (ongoing) - Provide monthly engagement and outcome reports to stakeholders
Barrier: Ineffective processes		
	Maintenance and Evolution	Audit and provide feedback Facilitate relay of clinical data to providers - Evaluate project to determine to scale project, continue in one location, or abandon project at the site - Present patient engagement and outcomes to all stakeholders
Communicate outcomes and progress to stakeholders	Implementation	Create a learning collaborative Remind clinicians - Utilize a multidisciplinary team to plan agenda, develop presentations, schedule, and promote/market activity
Goal: Ensure knowledge and information about the NDP is readily available		
Barrier: Information silos	Maintenance and Evolution	Create a learning collaborative - Utilize a multidisciplinary team to plan agenda, develop presentations, schedule, and promote/market activity Remind clinicians - Assess value and time required for the offering and determine continued feasibility
Adapt processes for NDP to adapt to participant needs by addressing their barriers	Implementation	Intervene with patients/consumers to enhance uptake and adherence - Assess engagement and patient volumes
Goal: Promote participant engagement	Maintenance and Evolution	Intervene with patients/consumers to enhance uptake and adherence - Assess engagement and patient volumes
Barrier: Low NDP participant engagement		

a ERIC: Expert Recommendations for Implementing Change.

b TTS: Tobacco Treatment Specialist.

**Table 3:** Timeline of nicotine dependence program (NDP) strategies mapped to ERIC^a^ strategies by implementation phase .

NDP Strategy	REP^b^ Phase			
	Pre-condition 2016–2017 (18 Months)	Pre-implementation 2017–2019 (30 months)	Implementation 2020–2022 (36 months)^c^	Maintenance and Evolution 2023-present
Build the business case for NDP	Assess for readiness and identify barriers and facilitators Identify and prepare champions			
Consult with experts who have experience in implementing the NDP	Shadow other experts Revise professional roles Use an implementation advisor			
Identify the NDP as a system priority to gain buy-in of key individuals	Mandate change Obtain formal commitments	Conduct educational meetings Stage implementation scale up		
Prioritize NDP as a part of the system's larger organizational goal	Mandate change	Mandate change Stage implementation scale up		
Utilize leadership across multiple systems (board of directors, executive/senior leadership, pharmacy, behaviroral health, surgery, cardiology)	Involve executive boards Prepare champions	Promote network weaving	Inform local opinion leaders	Inform local opinion leaders
Form multidisciplinary team consisting of VPs of product lines, IT, Finance, and essential medical personnel		Develop a formal implementation blueprint Promote network weaving		
Negotiate resources for funding, workforce, IT support		Access new funding Use advisory boards and workgroups	Tailor strategies	
Integrate NDP referrals/documentation into the EHR		Change record systems Promote adaptability	Promote adaptability	Promote adaptability
Reduce inefficiencies and implement process improvement		Develop and organize quality monitoring programs	Promote adaptability Purposely reexamine the implementation	
Develop standardized training and education materials		Develop educational materials Make training dynamic	Develop educational materials Make training dynamic	Develop educational materials Make training dynamic
Imbed NDP clinicians into offices/clinics they already work for		Create new clinical teams Revise professional roles		Revise professional roles
Offer NDP specific training to clinicians across the system		Conduct educational meetings Conduct educational outreach	Conduct educational meetings Conduct educational outreach	Conduct educational meetings Conduct educational outreach
Identify opportunities to expand and sustain program		Conduct cyclical small tests of change	Stage implementation scale up	Stage implementation scale up
Collect and interpret data supported by the EHR		Develop and organize quality monitoring systems	Audit and provide feedback Facilitate relay of clinical data to providers	Audit and provide feedback Facilitate relay of clinical data to providers
Communicate outcomes and progress to stakeholders			Create a learning collaborative Remind clinicians	Create a learning collaborative Remind clinicians
Adapt processes for NDP to adapt to participant needs by addressing their barriers			Intervene with patients/consumers to enhance uptake and adherence	Intervene with patients/consumers to enhance uptake and adherence

a ERIC: Expert Recommendations for Implementing Change.

b REP: Replicating Effective Programs.

c The COVID-19 Pandemic influenced the timing of activities during the Implementation Phase.

Key strategies utilized in the pre-condition phase included, *assessing for readiness and identifying barriers and facilitators to implementation* and *mandating change.* To do this, the implementation leader began conducting interviews to understand the current and historical tobacco work across the system. This was noted as a key strategy to get leadership support and approval to develop and pilot a tobacco treatment program to demonstrate feasibility and scalability of the new services. Activities in this phase began in 2016 and took approximately eighteen months to complete.

In the pre-implementation phase strategies including securing a *mandate for change* from system leadership to ensure that the NDP was prioritized and given necessary resources. Other strategies in included *developing a formal implementation blueprint, use of advisory boards and workgroups, changing record systems, developing educational materials, and conducting educational meetings and outreach.* Activities in this phase focused on ensuring that there was a sufficient and well-trained workforce and effective tools in place to implement the evidence-based tobacco treatment program. The pre-implementation phase lasted from 2017 to 2019 and spanned approximately 30 months.

The implementation phase began in early 2020 and focused on *informing local opinion leaders* about the NDP to promote awareness and engagement in the new standardized program. The primary focus early in the program was on employees and the patient population in one centralized location. Activities during this period focused on launching services and then *conducting educational meetings* with clinical teams to encourage them to refer patients, as well as marketing the employee program. The onset of the COVID-19 pandemic led to the need to adapt the original NDP model to allow for telehealth or virtual sessions. While this adaptation was responsive rather than proactive, it was used to *intervene with patients/consumers to enhance uptake and adherence.* This phase lasted for approximately 36 months, which was longer than anticipated given disruptions related to the onset of COVID-19.

The NDP moved into the maintenance and evolution phase beginning in 2023. This phase has been focused on *promoting adaptability* by identifying and training additional providers (i.e., pharmacists) to enhance reach*,* conducting *audits and providing feedback* to evaluate the program and make decisions to scale or abandon components, and *facilitating the relay of clinical data to providers* to inform stakeholders about patient engagement and outcomes related to the NDP. These strategies were identified as key factors to ensure the sustainability of the NDP beyond support from external funds.

## 4 Discussion

This study highlights the successful implementation of the CHNw Nicotine Dependence Program, with findings from interviews and CSAT demonstrating convergence across multiple factors. Qualitative interviews emphasized the pivotal role of inner setting factors, such as a patient-centered culture, strong leadership engagement, and alignment with existing workflows, in creating a supportive environment for implementation. These findings were also echoed in the CSAT responses, with a high overall rating indicating a relatively high perception of the sustainability potential for the NDP. Leadership engagement stood out as a critical factor across all phases, with executive leadership securing resources, integrating the program into strategic priorities, and addressing challenges such as workforce constraints and EHR integration. Again, this was reflected in the CSAT findings, with *engaged staff and leadership* receiving an average rating of 6.5 out of 7. The health system's equity-driven, patient-centered culture further facilitated adoption, with interviewees consistently describing a shared commitment to holistic care and reducing patient barriers to treatment aligning with an average CSAT rating of 6.6 out of 7.0 for how well the practice fits in the culture of the team. These findings highlight the importance of a supportive innovation climate, clear leadership roles, and strong leader-member relationships in fostering readiness and successful implementation (20–22). Additionally, the program's compatibility with existing workflows minimized disruption and enhanced usability, underscoring the importance of EHR integration and IT support in sustaining tobacco treatment interventions (23–25).

A key contribution of this study is our use of a multi-methods design drawing on established implementation frameworks to retrospectively reconstruct implementation processes and map strategies, aligned with contextual conditions, across phases of implementation. This approach provides an example of how practice-based evidence can be used to provide actionable insights to inform the implementation, adaptation, and evaluation of evidence-based interventions (8–11). Further, by mapping ERIC-defined implementation strategies across REP phases, this study extends existing work by demonstrating how strategies can be sequenced over time (26, 27). Lastly, the integration of the sustainability assessment data highlights the connection between implementation processes and long-term organizational capacity, reinforcing the importance of aligning strategies with both context and sustainment goals. Together, these contributions extend existing literature by bridging practice and theory, and by producing a pragmatic, phase-specific guide for health systems seeking to implement and sustain evidence-based tobacco treatment programs.

### 4.1 Limitations

It is important to put the findings and contributions of this study in context with its limitations. First, we utilized a purposive sample for both interviews and CSAT and responses to both relied on self-report, which may introduce social desirability and recall bias. This may be reflected in the primarily positive responses identified through both interview and CSAT data. Additionally, strategy mapping was conducted retrospectively, which may affect the precision of sequencing; however, inclusion of the implementation lead, and document review helped mitigate this concern. Lastly, the study was conducted within a single health system, which may limit generalizability. Despite these limitations, our findings underscore that sustainable systems change is not merely about adopting external best practices but about optimizing locally driven programs rooted in context, shaped by frontline expertise, and refined through iterative feedback. The use of established frameworks such as REP, ERIC, and CFIR enhances the transferability of findings and provides a structured blueprint that other health systems can adapt when developing and implementing evidence-based tobacco treatment programs.

## 5 Conclusion

As tobacco use continues to pose a serious public health threat, scaling context-responsive, practice-informed models can assist in achieving equitable and lasting improvements in tobacco treatment delivery. One of this study's primary contributions is the temporal mapping of ERIC strategies across REP phases, offering a practical framework for sequencing implementation efforts in real-world systems. The resulting implementation blueprint offers a transferable process for other health systems to identify, organize, and adapt strategies to support the integration and sustainment of evidence-based tobacco treatment interventions. Future research should focus on assessing how this model can be scaled to support other health systems in integrating tobacco treatment into routine practice.

## Data Availability

Aggregate, de-identified data can be provided upon reasonable request, further queries can be directed to the corresponding author.

## Supplementary material

Supplementary Data Sheet 1. Interview Guide 6.

Supplementary Data Sheet 2. Interview Guide 5.

Supplementary Data Sheet 3. Interview Guide 4.

Supplementary Data Sheet 4. Interview Guide 3.

Supplementary Data Sheet 5. Interview Guide 2.

Supplementary Data Sheet 6. Interview Guide 1.
