# Supplementary material for: Practice-informed implementation: a multi-method approach to map strategies for sustainable tobacco treatment integration within healthcare settings
Source: Front Health Serv. 2026 Aug 4;6:1749305. doi: 10.3389/frhs.2026.1749305 (PMC13481515; doi:10.3389/frhs.2026.1749305)
Supplement: Supplementary file 2 [file Datasheet2.docx]

**Community Health Network Case Study Interview Guide- IT/Data Analyst**

**Introduction**

*Thank you for participating. As you may recall from our request for this telephone interview, we are from the IU Fairbanks School of Public Health, and are part of the Health Systems Change evaluation team funded by Indiana Tobacco Prevention and Cessation. The purpose of today’s interview is to understand the process by which the Community Health Network has implemented a tobacco treatment initiative. This project has been approved by the Institutional Review Board at Indiana University.*

*If we have your permission, we would like to record this interview for note-taking purposes. This recording will be destroyed after the conclusion of this study. The information you share with us today will be used to summarize experiences with implementing this tobacco treatment initiative. Your name and any information that would identify you will not be shared in any reports or findings. Further, any information that you share that may refer to specific employees or similar parties will be deidentified to protect the anonymity of all parties. Finally, you may withdraw from this interview at any time, and you will not face any negative repercussions. Should you withdraw from this study, any associated notes and recordings will be destroyed immediately. Do we have your consent to participate and record this interview?*

**Background**

1. Could you explain your role and how you are involved or engaged in the implementation or delivery of the Nicotine Dependence Program at Community Health Network?
2. How long have you been in this role?

**Intervention Characteristics**

*Planning* ***(Process)***

1. What was done to get a plan in place to implement EHR changes to support the NDP?
2. What was your role in the planning process?

- *Adaptability*

1. Were there any changes that were made to the original intervention??
2. What was the process for deciding whether changes are/were needed in the EHR to support the NDP so that it works well within CHNw?

*Trialability*

1. Was the NDP workflow piloted prior to full-scale implementation? If so, how did it inform the final implementation?

*Complexity*

1. How complicated was the NDP workflow to implement? How did it compare to other workflow implementations you’ve been involved with?

*Costs*

1. What costs were considered when deciding to implement the NDP?
2. What costs were incurred to implement the NDP?

**Inner Setting**

*Structural Characteristics*

1. How has the infrastructure of your organization (social architecture, age, maturity, size, or physical layout) affected the implementation of the NDP?
2. Were any infrastructure changes needed to accommodate the NDP and the new workflows?
   - This could include changes in scope of practice, formal policies, information systems or electronic records systems.

*Culture*

1. How do you think your organization's culture (general beliefs, values, assumptions that people embrace) affected the implementation of the NDP?
   - Can you describe an example that highlights this?

*Implementation Climate*

1. What was the general level of receptivity in your organization/unit to implementing the NDP?

*Compatibility*

1. Can you describe how the NDP was integrated into existing processes?
   - How did it interact or conflict with existing programs or processes?
2. How well did the NDP fit with existing work processes and practices in CHNw?

*Reflecting & Evaluating* ***(Process)***

1. What kind of information is collected about the NDP workflows as part of the Dashboard/Smart Form?
   - Which measures are tracked? How are they tracked?

*Opinion Leaders* ***(Process)***

1. Who were the key influential individuals to get on board with this implementation?

*Champions* ***(Process)***

1. Other than the formal implementation leader, were there people in your organization that have championed (go above and beyond what might be expected) the NDP?
   - If yes, who were/are these champions? How did they get involved with the implementation? How did they champion the NDP?

*Leadership Engagement*

1. What level of endorsement or support have you seen or heard from leaders?
