# Supplementary material for: Practice-informed implementation: a multi-method approach to map strategies for sustainable tobacco treatment integration within healthcare settings
Source: Front Health Serv. 2026 Aug 4;6:1749305. doi: 10.3389/frhs.2026.1749305 (PMC13481515; doi:10.3389/frhs.2026.1749305)
Supplement: Supplementary file 3 [file Datasheet3.docx]

**Community Health Network Case Study Interview Guide- Leadership**

**Introduction**

*Thank you for participating. As you may recall from our request for this telephone interview, we are from the IU Fairbanks School of Public Health, and are part of the Health Systems Change evaluation team funded by Indiana Tobacco Prevention and Cessation. The purpose of today’s interview is to understand the process by which the Community Health Network has implemented a tobacco treatment initiative. This project has been approved by the Institutional Review Board at Indiana University.*

*If we have your permission, we would like to record this interview for note-taking purposes. This recording will be destroyed after the conclusion of this study. The information you share with us today will be used to summarize experiences with implementing this tobacco treatment initiative. Your name and any information that would identify you will not be shared in any reports or findings. Further, any information that you share that may refer to specific employees or similar parties will be deidentified to protect the anonymity of all parties. Finally, you may withdraw from this interview at any time, and you will not face any negative repercussions. Should you withdraw from this study, any associated notes and recordings will be destroyed immediately. Do we have your consent to participate and record this interview?*

**Background**

1. Could you explain your role and how you are involved or engaged in the implementation or delivery of the Nicotine Dependence Program at Community Health Network?

**Intervention Characteristics**

*Intervention Source*

1. Who developed the Nicotine Dependence Program (NDP)?
2. Why was the NDP implemented in CHNw?
   - Who decided to implement the NDP?
   - How was the decision made to implement the NDP?

*Relative Advantage*

1. Before the NDP was implemented, were there similar existing programs?
   - If so, how does the NDP compare to other similar existing programs?
   - What advantages does the NDP have compared to existing programs?
   - What disadvantages does the NDP have compared to existing programs?

*Planning* ***(Process)***

2. What was done to get a plan in place to implement the NDP?
3. What was your role in the planning process?

*Complexity*

1. How complicated was the NDP to implement? How did it compare to other intervention implementations you’ve been involved with from a leadership perspective?

*Costs*

1. What costs were considered when deciding to implement the NDP?
2. What costs were incurred to implement the NDP?

**Outer Setting**

*Patient Needs & Resources*

1. How were these needs and preferences of individuals served by CHNw considered when implementing the NDP?
2. How well do you think the NDP meets the needs of the individuals served by your organization?

**Inner Setting**

*Structural Characteristics*

1. How has the infrastructure of your organization (social architecture, age, maturity, size, or physical layout) affected the implementation of the NDP?
2. Were any infrastructure changes needed to accommodate the NDP?
   - This could include changes in scope of practice, formal policies, information systems or electronic records systems.

*Culture*

1. How do you think your organization's culture (general beliefs, values, assumptions that people embrace) affected the implementation of the NDP?
   - Can you describe an example that highlights this?

*Implementation Climate*

1. What was the general level of receptivity in your organization to implementing the NDP?

*Tension for Change*

1. How did the NDP fill gaps or needs of CHNw and/or the patients you serve?

*Compatibility*

1. How well does the NDP fit with your values and norms and the values and norms within the organization?

*Organizational Incentives & Rewards*

1. Were any incentives placed to ensure that the implementation of the NDP was successful?

*Available Resources*

1. Do you feel there are sufficient resources to continue to implement and administer the NDP?
