# Supplementary material for: Practice-informed implementation: a multi-method approach to map strategies for sustainable tobacco treatment integration within healthcare settings
Source: Front Health Serv. 2026 Aug 4;6:1749305. doi: 10.3389/frhs.2026.1749305 (PMC13481515; doi:10.3389/frhs.2026.1749305)
Supplement: Supplementary file 6 [file Datasheet6.docx]

**Community Health Network Case Study Interview Guide- Dedicated NDP Staff**

**Introduction**

*Thank you for participating. As you may recall from our request for this telephone interview, we are from the IU Fairbanks School of Public Health, and are part of the Health Systems Change evaluation team funded by Indiana Tobacco Prevention and Cessation. The purpose of today’s interview is to understand the process by which the Community Health Network has implemented a tobacco treatment initiative. This project has been approved by the Institutional Review Board at Indiana University.*

*If we have your permission, we would like to record this interview for note-taking purposes. This recording will be destroyed after the conclusion of this study. The information you share with us today will be used to summarize experiences with implementing this tobacco treatment initiative. Your name and any information that would identify you will not be shared in any reports or findings. Further, any information that you share that may refer to specific employees or similar parties will be deidentified to protect the anonymity of all parties. Finally, you may withdraw from this interview at any time, and you will not face any negative repercussions. Should you withdraw from this study, any associated notes and recordings will be destroyed immediately. Do we have your consent to participate and record this interview?*

**Background**

1. Could you explain your role and how you are involved or engaged in the implementation or delivery of the Nicotine Dependence Program at Community Health Network?
2. How long have you been involved with this program?

**Intervention Characteristics**

*Intervention Source*

1. Who developed the NDP?
2. Why was the Nicotine Dependence Program implemented in your setting?
   - Who decided to implement the NDP?

*Relative Advantage*

1. Before the NDP was implemented, were there similar existing programs?
   - If so, how does the NDP compare to other similar existing programs in your setting?
   - What advantages does the NDP have compared to existing programs?
   - What disadvantages does the NDP have compared to existing programs?
2. Were there other interventions that were suggested?
   - If so, what were those interventions? Why was this intervention decided upon? Can you describe that intervention?
   - Why would people prefer the alternative?

*Adaptability*

1. Were there any changes that were made to the original intervention, so it worked for your setting?
2. What was the process for deciding whether changes are needed to the NDP so that it works well in your setting?

*Trialability*

1. Was the NDP piloted prior to full-scale implementation? If so, how did it inform the final implementation?

*Complexity*

1. How complicated was the NDP to implement? How did it compare to other intervention implementations you’ve been involved with?

**Outer Setting**

*Patient Needs & Resources*

1. How were these needs and preferences of individuals served by CHNw considered when implementing the NDP?
2. How well do you think the NDP meets the needs of the individuals served by your organization?

*Intervention Participants* ***(Process)***

3. How do you or your colleagues communicate to the individuals that are served by your organization about the NDP?

*Patient Needs & Resources*

4. How have the individuals served by your organization responded to the NDP?
5. What barriers do individuals served by your organization face to participating in the NDP?

**Inner Setting**

*Structural Characteristics*

1. How has the infrastructure of your organization (social architecture, age, maturity, size, or physical layout) affected the implementation of the NDP?
2. Were any infrastructure changes needed to accommodate the NDP?
   - This could include changes in scope of practice, formal policies, information systems or electronic records systems.

*Culture*

1. How do you think your organization's culture (general beliefs, values, assumptions that people embrace) affected the implementation of the NDP?
   - Can you describe an example that highlights this?

*Implementation Climate*

1. What was the general level of receptivity in your organization to implementing the NDP?

*Tension for Change*

1. How did the NDP fill gaps or needs?

*Compatibility*

1. How well does the NDP fit with your values and norms and the values and norms within the organization?
2. Can you describe how the NDP was integrated into existing processes?
   - How did it interact or conflict with existing programs or processes?
3. How well did the NDP fit with existing work processes and practices in your setting?

*Organizational Incentives & Rewards*

1. Were any incentives placed to ensure that the implementation of the NDP was successful?

*Goals & Feedback*

1. Have you/your unit/your organization set goals related to the implementation of the NDP?
   - [If yes] What are the goals?
2. To what extent are organizational goals monitored for progress?
3. How does implementation of the NDP align with other organizational goals?

*Reflecting & Evaluating* ***(Process)***

1. What kind of information is collected about the NDP?
   - Which measures are tracked? How are they tracked? How is this information

*Opinion Leaders* ***(Process)***

1. Who were the key influential individuals to get on board with this implementation?

*Champions* ***(Process)***

1. Other than the formal implementation leader, were there people in your organization have championed (go above and beyond what might be expected) the NDP?
   - If yes, who were/are these champions? How did they get involved with the implementation? How did they champion the NDP?

*External Change Agents* ***(Process)***

1. Were any individuals or teams outside your organization involved with implementing the NDP?
   - Who were these people?
   - How did they get involved?
   - What were their roles/activities in implementation?

*Key Stakeholders* ***(Process)***

1. What steps have been taken to encourage individuals to commit to using the NDP?
2. What is your communication or education strategy for getting the word out about the NDP?

*Leadership Engagement*

1. What level of endorsement or support have you seen or heard from leaders?

*Available Resources*

1. Are there sufficient resources to implement and administer the NDP?

*Access to Knowledge & Information*

1. What kind of training was planned to educate staff and providers about the implementation? Was this sufficient?

What ongoing training is provided to staff delivering the implementation?
