# Supplementary material for: Practice-informed implementation: a multi-method approach to map strategies for sustainable tobacco treatment integration within healthcare settings
Source: Front Health Serv. 2026 Aug 4;6:1749305. doi: 10.3389/frhs.2026.1749305 (PMC13481515; doi:10.3389/frhs.2026.1749305)
Supplement: Supplementary file 7 [file Table1.docx]

Supplementary Material

# 1 Supplementary Figures and Tables

## 1.1 Supplementary Figures

**Figure.** **Workflow of the Community Health Network Nicotine Dependence Program (NDP).** This figure depicts the structured workflow of the CHNw Nicotine Dependence Program, delivered over approximately eight visits. The intervention begins with an intake visit focused on assessment and motivational interviewing, followed by a motivation phase to enhance engagement. Patients then develop individualized quit plans, including setting a quit date and identifying triggers. The final phase emphasizes quit support and relapse prevention. An additional support component allows ongoing engagement beyond the core program, reflecting a flexible, patient-centered approach to tobacco cessation.

*Note: CHNw’s process may also include the provision of tobacco treatment medication to patients. The evidence-based tobacco treatment algorithm is proprietary to CHNw and can be provided upon request.*

## 1.2 Supplementary Tables

**Table 1. Type of interviewee with role descriptions and number per type (n=15)**

| **Type of Interviewee** | **Role Description** | **N** |
| --- | --- | --- |
| Hospital Leadership | Executive and administrative leadership | 3 |
| Dedicated NDP Staff | Tobacco treatment specialists, implementation lead | 3 |
| Internal Champions | Pharmacy, psychology, and clinical champions | 3 |
| Referring Providers | Clinical referral sources | 2 |
| Internal Technical Assistance | IT/data and quality improvement support | 2 |
| External Technical Assistance | External consultants and program developers | 2 |

**Table 2. Description of documents used in document review including examples and purpose**

| **Document Category** | **Examples** | **Purpose** |
| --- | --- | --- |
| Strategic Planning Documents | Business plan/implementation plans | Program development and organizational goals |
| Clinical Policies & Protocols | NDP policy, treatment protocols, medication algorithms | Clinical service delivery |
| Workflow Documents | Appointment flowcharts, care pathways | Patient workflow and care processes |
| Quality Improvement Materials | Process improvement plan | Implementation and process improvement |
| Training Materials | Provider manuals and onboarding resources | Workforce development |
